# Effect of dialysate sodium concentration on intradialytic blood pressure variability and related clinical outcomes: A single-center prospective cohort study

**DOI:** 10.1097/MD.0000000000048667

**Published:** 2026-05-08

**Authors:** Ferah Taran, Hayriye Sayarlioglu

**Affiliations:** a Department of Nephrology, Sirnak State Hospital, Sirnak, Türkiye; b Department of Nephrology, Faculty of Medicine, Ondokuz Mayis University, Samsun, Türkiye.

**Keywords:** blood pressure variability, dialysate sodium, hemodialysis, interdialytic weight gain, ultrafiltration

## Abstract

Dialysate sodium concentration (dNa) is a key determinant of hemodynamic stability during hemodialysis. However, the clinical relevance of small reductions in dNa remains uncertain, particularly regarding intradialytic blood pressure variability and ultrafiltration efficiency. This study aimed to evaluate the effect of reducing dialysate sodium from 140 mmol/L to 138 mmol/L on intradialytic systolic and diastolic blood pressure variability, interdialytic weight change, and ultrafiltration adequacy. This prospective, single-center cross-over study included ten adult hemodialysis patients (N = 10) treated sequentially with dNa concentrations of 140 and 138 mmol/L for 2 consecutive months (12 sessions per phase). Paired comparisons were performed using Wilcoxon signed-rank tests. No significant differences were observed between 140 mmol/L and 138 mmol/L dialysate sodium in systolic or diastolic BP variability, interdialytic weight gain, intradialytic weight loss, or ultrafiltration adequacy (all *P* > .05). Median systolic BP variability was 15.9 mm Hg (IQR 13.0–18.2) vs 14.3 mm Hg (IQR 12.4–18.4) (HL diff 2.00 [95 % CI–7.44, 6.53]); diastolic BP variability 7.9 mm Hg (IQR 6.8–11.0) vs 7.9 mm Hg (IQR 7.7–8.8) (HL diff–0.15 [95 % CI–3.90, 3.35]). Exploratory subgroup trends (greater systolic variability in females, lower in CAD patients under 138 mmol/L) were not statistically reliable due to small group sizes. A modest 2 mmol/L reduction in dialysate sodium was not associated with clear short-term changes in hemodynamic or fluid-related effects. Larger multicenter studies are needed to clarify the clinical impact of individualized sodium prescriptions.

## 1. Introduction

Cardiovascular diseases are the leading cause of mortality in hemodialysis patients; this condition is often closely related to volume overload and uncontrolled blood pressure.^[1–3]^ Significant fluctuations in blood pressure may occur during hemodialysis. Intradialytic hypotension (IDH) is one of the most common complications of chronic HD, occurring in 7% to 50% of sessions.^[4,5]^ Each IDH episode causes transient organ hypoperfusion that may lead to arrhythmia, ischemic injury, or vascular access thrombosis, and repeated episodes increase long-term mortality risk.^[6–8]^ Conversely, 5% to 15% of patients experience paradoxical intradialytic hypertension (IDHT), which is associated with 30% to 40% higher cardiovascular mortality.^[7–9]^ These contrasting hemodynamic patterns highlight the importance of maintaining stable blood pressure during dialysis, and recent consensus reports emphasize that such stability depends not only on ultrafiltration or volume status but also on diffusive sodium shifts across the dialysis membrane.^[10]^

In order to reduce the volume load and stabilize the intradialytic blood pressure profile, the sodium concentration of the dialysate fluid stands out as a modifiable parameter in the dialysis prescription.^[11,12]^ Currently, most centers prefer to use a fixed dialysate containing ~137 to 140 mmol/L sodium in hemodialysis; however, if the dialysate dNa is higher than the patient’s plasma sodium, sodium is transferred to the patient by diffusion, and if it is lower, sodium is removed from the patient. Adjusting the dialysate sodium level has significant effects on the patient’s fluid balance and blood pressure. For example, high-sodium dialysis can reduce the development of hypotension during the session by supporting osmotic balance; however, it is also associated with sodium loading, excessive thirst, increased interdialytic weight gain, and elevated blood pressure.^[13,14]^ Conversely, using a lower dNa can reduce the patient’s net sodium-water retention and slightly lower blood pressure; however, this approach also carries the risk of more frequent intradialytic hypotension episodes.^[1,15,16]^ It has also been reported that clinical effects become more pronounced as the difference in dialysate sodium levels increases. For example, when dialysis was performed with 141 mmol/L sodium instead of 137 mmol/L, patients’ 72-hour ambulatory blood pressure was shown to be significantly higher (mean 72-hour systolic blood pressure (SBP) increased from ~125 to ~132 mm Hg).^[10]^

However, available studies mostly examined broad sodium gradients (≥3–5 mmol/L), whereas minor adjustments (e.g., 2 mmol/L) have been largely overlooked. It remains uncertain whether such small decrements could produce measurable hemodynamic effects or improve intradialytic stability without compromising ultrafiltration tolerance.^[17]^

Another approach aimed at maintaining hemodynamics is the use of “sodium profiling” techniques, which involve gradually changing the sodium concentration of the dialysate during the session. This approach, which involves a gradual decrease from a higher sodium level at the start of dialysis, has been found to be successful in increasing intravascular volume and reducing the development of hypotension and cramps.^[13,18–21]^ However, due to side effects such as excessive thirst and weight gain associated with additional sodium load, sodium profiling is not widely used in routine practice today.^[13]^ Overall, data on the impact of dialysate sodium optimization on long-term patient outcomes are conflicting. In recent large patient series, it has been suggested that consistently high or low-sodium dialysis regimens do not provide a significant advantage in terms of long-term “hard” outcomes or dialysis adequacy.^[17,22–24]^ Data on the long-term effects of fixed sodium levels are limited and conflicting; therefore, the need for randomized controlled trials is frequently emphasized in the literature.^[10,12,23,25–27]^ In particular, recent meta-regressions and registry-based analyses have underscored that individualized sodium prescription – rather than a universal fixed concentration – may better balance intradialytic tolerance and cardiovascular risk.^[23]^

It is noteworthy that most of the studies in the literature are short-term (median follow-up period is only ~4 weeks) and some of them were performed using dialysis techniques that are no longer routinely used. Moreover, few reports have provided quantitative assessment of intra-session blood pressure variability (BPV), which is now recognized as an independent cardiovascular risk marker.^[15]^ Prospective data that quantitatively examines BPV (e.g., intra-session and inter-session variations in systolic and diastolic pressure values) and evaluates this in conjunction with ultrafiltration success is extremely limited. To address these information gaps, the planned study prospectively compared 2 different dialysate sodium levels (138 and 140 mmol/L) in the same patient group over consecutive sessions. The specific comparison between 140 and 138 mmol/L was deliberately chosen to test whether a minimal 2 mmol/L reduction – representing a feasible, low-risk adjustment in clinical practice – would produce detectable differences in hemodynamic variability and volume management. In this single-center, 1-sequence cross-over study, each individual received both sodium levels sequentially (140 mmol/L followed by 138 mmol/L), and intradialytic BPV as well as the rate of achieving the target ultrafiltration volume were evaluated comparatively. The primary objective was to determine the effects of dNa concentration (138 and 140 mmol/L) on BPV and ultrafiltration adequacy during hemodialysis.

## 2. Materials and methods

### 2.1. Study design and center

We conducted a single-center, prospective, within-subject study to compare the effects of 2 different dialysate sodium concentrations on hemodynamics and fluid management in hemodialysis patients. The study was conducted at the Hemodialysis Unit of the Department of Nephrology at Sirnak State Hospital between June 1 and July 31, 2025. The study was conceived as a pilot and exploratory investigation due to the limited eligible patient pool at the center. A formal sample size calculation was not feasible; however, with ten paired observations (N = 10), the design was powered to detect only moderate-to-large within-subject differences (Hodges–Lehmann median difference ≈ 5–6 mm Hg) in SBPV.

### 2.2. Participants

**Figure 1.**
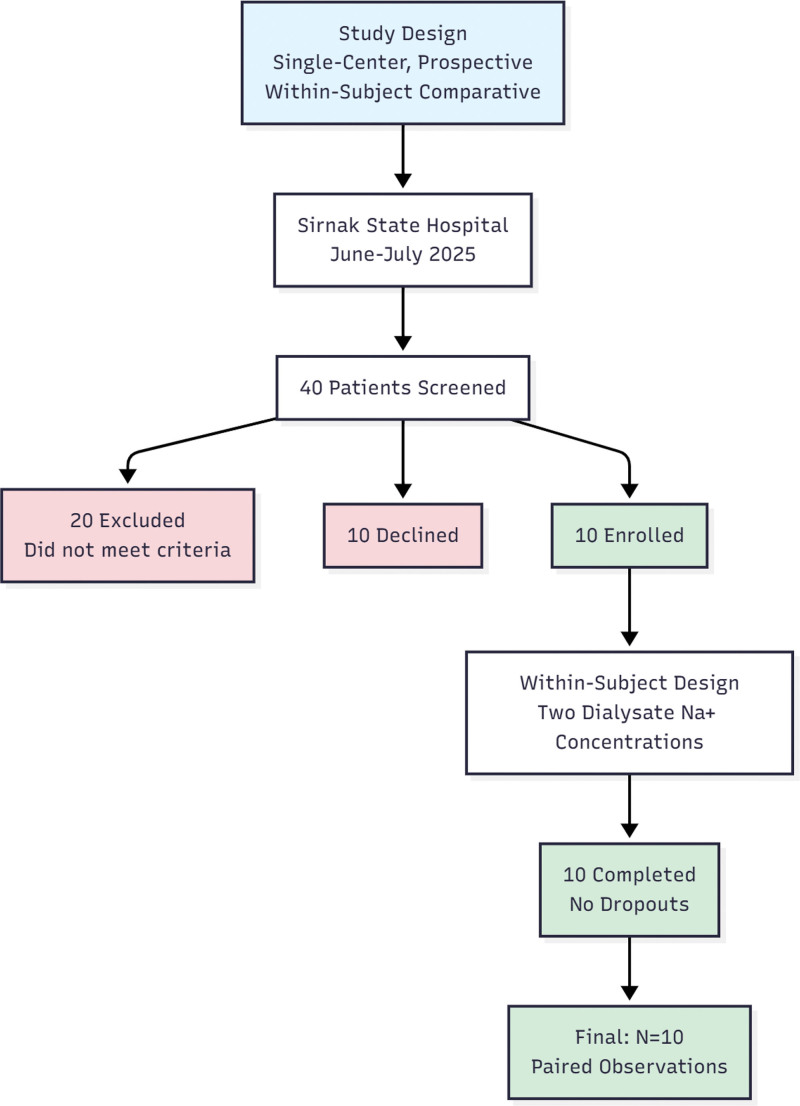
Study flow diagram.

The study included adult patients aged 18 years and older who had been receiving regular hemodialysis treatment 3 times a week for at least 3 months. Exclusion criteria: malignancy, adrenal insufficiency, untreated hypothyroidism, uncontrolled diabetes (fasting glucose ≥ 300 mg/dL), orthostatic hypotension, heart failure with EF ≤ 40%, cardiovascular event within the past 3 months, chronic liver disease, use of centrally acting medications associated with hyponatremia, active infection, and hemoglobin < 8 g/dL. Of the 40 patients screened for eligibility, 20 did not meet inclusion criteria (e.g., cardiovascular event within the past 3 months, heart failure, or infection) and 10 declined participation. The remaining 10 patients were enrolled and completed all study sessions without dropout. A STROBE flow diagram summarizing recruitment and retention is presented in Figure 1.

### 2.3. Intervention and protocol

All patients received dialysate containing a constant dNa of 140 mmol/L during the first month, which was reduced to 138 mmol/L in the second month. Each individual was considered their own control. Sessions were conducted using (Fresenius 4008S) and high-flux dialyzers (Fresenius FX 80), and standard treatment protocols were applied for all patients.

Pre- and post-session body weights were measured using calibrated electronic scales, while blood pressure and heart rate were measured at baseline, every hour, and at the end of the session using automatic noninvasive monitors. Hypotension was defined as symptomatic and SBP ≤ 90 mm Hg or drops requiring intervention. Nausea, cramps, the need for more than 100 mL of saline infusion, and session terminations were recorded.

During treatment, sodium bicarbonate and dialysate calcium concentrations were kept constant. The number of antihypertensive medications was intended to be maintained; however, dose adjustments or discontinuations were made in patients whose clinical condition improved with the change in dNa. Limited interventions performed for clinical reasons (e.g., saline administration, Na adjustment) were prospectively documented during each session and included in the analysis. Parameters other than dNa in the dialysate (e.g., Ca, bicarbonate) were kept constant. Medication changes were monitored weekly, the number of antihypertensive medications was documented in clinical records, and evaluated as a control variable in the analysis.

No interventions were made except for adverse events, and the same protocol was applied to all patients. This situation was controlled by excluding sessions outside the protocol from the analysis in order to minimize possible deviations.

Data quality assurance included double entry of session data and cross-checking against dialysis machine printouts. Any implausible records (e.g., interdialytic weight change < –10% or > +10%) were verified from source charts; if verification was not possible, the record was excluded according to the predefined protocol.

### 2.4. Outcome measures

The primary outcome was the effect of dialysate sodium concentration on intradialytic BPV and inadequate ultrafiltration (IAUF). BPV was defined as the intra-session standard deviation of systolic and diastolic blood pressure measurements obtained at baseline, hourly, and at session completion. Inter-session variability was not included in this analysis. Secondary outcomes included interdialytic weight gain (IDWG), intradialytic weight loss (IDWL), number of hypotensive events, need for additional dialysis, need for saline infusion, frequency of cramps, and the relationship between BPV and clinical-biochemical markers. IDWG and IDWL were calculated as percentage changes relative to post-dialysis dry weight:


$$\begin{array}{l} IDWG\;\left(\%\right)=\frac{\left(Pre\text{-}HD-Previous\;Post\text{-}HD\right)}{Dry\;Weight}\times 100 \end{array}$$

$$\begin{array}{l} IDWL\;\left(\%\right)=\frac{\left(Pre\text{-}HD-Post\text{-}HD\right)}{Dry\;Weight}\times 100 \end{array}$$

## 3. Statistical analysis

All statistical analyses were conducted using IBM SPSS Statistics v25.0 (IBM Corp., Armonk). The normality of continuous variables was assessed using the Kolmogorov–Smirnov test. Non-normally distributed data were presented as median (minimum–maximum). Primary endpoints (SBP-BPV and DBP-BPV) were compared between dNa 140 mmol/L and 138 mmol/L using the Wilcoxon signed-rank test, with Hodges–Lehmann median difference and 95% confidence intervals reported. Holm–Bonferroni correction was applied for multiple comparisons of primary outcomes. Secondary endpoints (IDWG, IDWL, IAUF, etc) were analyzed with Wilcoxon or Kruskal–Wallis tests as appropriate, using Benjamini–Hochberg false discovery rate control (q = 0.05) to limit type I error. Exploratory correlation analyses were also performed between BPV metrics and markers of volume management (ultrafiltration shortfall, interdialytic weight gain, intradialytic weight loss) using Spearman rho.

Given the pilot nature of the study and the limited number of paired observations (N = 10), no formal a priori sample size calculation was performed. However, based on the observed within-subject variability of SBPV (SD approximately 6–7 mm Hg, consistent with prior cross-over studies), the study had approximately 80% power to detect a paired difference of about 5 to 6 mm Hg at a 2-sided α level of 0.05. Accordingly, nonsignificant findings should be interpreted as an inability to exclude small effect sizes (<3 mm Hg) rather than evidence of equivalence.

Subgroup comparisons (sex, vascular access, coronary artery disease) were considered exploratory and interpreted descriptively due to very small group sizes (n = 3–4 per cell). A 2-tailed *P* < .05 was considered statistically significant for unadjusted tests.

## 4. Ethical approval

The study received approval from the Health Sciences University Gazi Yasargil Training and Research Hospital Ethics Committee (Decision No: 458, dated April 25, 2025), and written informed consent was obtained from all participants.

## 5. Results

Table 1 presents descriptive statistics on baseline clinical parameters, session-related symptoms, and antihypertensive drug use for the study cohort (N = 10). Female patients represented 30% of the sample, and the majority had an arteriovenous fistula as their vascular access (90%). Residual diuresis >100 mL/day was present in 70% of patients, while comorbid diabetes mellitus and hypertension were observed in 30% and 90%, respectively. Coronary artery disease and cerebrovascular disease were present in 40% and 10% of the cohort, respectively. Session-related symptom analysis showed that, at dNa 138 mmol/L, 7 patients (70%) experienced at least 1 intradialytic symptom from the predefined list, whereas 3 patients (30%) remained symptom-free. At dNa 140 mmol/L, 3 patients (30%) reported symptoms and 7 (70%) reported none. During the 138 mmol/L period, the distribution of antihypertensive use was: 4 patients (40%) with no antihypertensive medication, 2 (20%) using 1 drug, and 4 (40%) using 2 or more. In the 140 mmol/L period, 2 patients (20%) used no antihypertensives, 4 (40%) used 1, and 4 (40%) used 2 or more agents.

**Table 1:** Baseline clinical characteristics, comorbidities, vascular access, residual diuresis, and antihypertensive medication burden of the study cohort (N = 10).

Variable (valid n = 10)	Category	*n*	%
Sex	Female	3	30.0
	Male	7	70.0
Vascular access	Catheter	1	10.0
	Fistula	9	90.0
Residual diuresis	Anuric	3	30.0
	>100 mL day–1	7	70.0
CKD etiology	Diabetic nephropathy	3	30.0
	Hypertensive nephropathy	4	40.0
	Unknown	3	30.0
Diabetes mellitus	Present	3	30.0
	Absent	7	70.0
Hypertension	Present	9	90.0
	Absent	1	10.0
Coronary artery disease	Present	4	40.0
	Absent	6	60.0
Cerebrovascular disease	Present	1	10.0
	Absent	9	90.0
Session symptoms (dNa 138 mmol/L)	Nausea/ vomiting ≥1	7	70.0
	None	3	30.0
Session symptoms (dNa 140 mmol/L)	Nausea/ vomiting ≥1	3	30.0
	None	7	70.0
Antihypertensive count (dNa 138 mmol/L)	0 drugs	4	40.0
	1 drug	2	20.0
	≥2 drugs	4	40.0
Antihypertensive count (dNa 140 mmol/L)	0 drugs	2	20.0
	1 drug	4	40.0
	≥2 drugs	4	40.0

CKD = chronic kidney disease, dNa = dialysate sodium concentration.

Median intradialytic systolic blood pressure (SBP) variability was 15.91 mm Hg (13.00–18.18) during 140 mmol/L dialysate sodium sessions and 14.31 mm Hg (12.40–18.38) during 138 mmol/L sessions, with a Hodges–Lehmann paired difference of 2.00 mm Hg (95% CI − 7.44 to 6.53). Similarly, diastolic BP variability remained stable between sodium levels (7.95 [6.82–11.04] vs 7.89 [7.68–8.75] mm Hg; HL diff − 0.15, 95% CI − 3.90 to 3.35). No consistent directional change was observed in fluid-related parameters. Interdialytic weight gain was 3.68% (3.39–4.67) under 140 mmol/L and 3.95% (3.32–4.64) under 138 mmol/L dialysate sodium, while intradialytic weight loss showed near-identical medians (3.59% vs 3.86%). *Ultrafiltration shortfall* remained minimal (median 0.00% vs 0.03%). Overall, the 2 mmol/L sodium reduction produced no systematic shift in intradialytic hemodynamic variability or fluid management parameters, suggesting comparable short-term tolerability and hemodynamic stability between 140 and 138 mmol/L dialysate prescriptions in this pilot cohort (Table 2).

**Table 2:** Intradialytic BPV and fluid-related outcomes under 140 mmol/L versus 138 mmol/L dialysate sodium (N = 10).

Outcome	140 mmol/L median (IQR)	138 mmol/L median (IQR)	Hodges–Lehmann difference (138 − 140)	95% CI	Adjusted *P*-value
Intradialytic systolic BP variability (SD, mm Hg)	15.91 (13.00–18.18)	14.31 (12.40–18.38)	2.00	(−7.44 to 6.53)	NA
Intradialytic diastolic BP variability (SD, mm Hg)	7.95 (6.82–11.04)	7.89 (7.68–8.75)	−0.15	(−3.90 to 3.35)	NA
Interdialytic weight gain (% of dry weight)	3.68 (3.39–4.67)	3.95 (3.32–4.64)	−0.17	(−0.98 to 0.91)	NA
Intradialytic weight loss (% of dry weight)	3.59 (3.23–4.26)	3.86 (3.28–4.54)	−0.03	(−0.51 to 1.31)	NA
Ultrafiltration shortfall (% of prescribed UF not achieved)	0.00 (0.00–0.27)	0.03 (0.00–0.12)	0.03	(−0.33 to 0.44)	NA

Data are presented as median (IQR). Hodges–Lehmann difference = median paired difference (138 − 140) with approximate 95% confidence interval. SBP and DBP variability were designated a priori as primary outcomes (Holm adjustment); fluid-related parameters were exploratory (Benjamini–Hochberg false discovery rate). Exact paired *P*-values could not always be computed due to uniform or near-uniform paired differences in this small cohort (N = 10); thus, interpretation emphasizes effect size and precision over hypothesis testing. Intradialytic BP variability reflects the within-session SD of systolic or diastolic BP. Interdialytic weight gain = [(pre-HD − previous post-HD)/ dry weight] × 100. Intradialytic weight loss = [(pre-HD − post-HD)/ dry weight] × 100. Ultrafiltration shortfall = proportion of prescribed ultrafiltration volume not achieved (IAUF). Implausible weight or UF values (< −10% or > +10%) were excluded as predefined quality control criteria.

HD = hemodialysis, IAUF = inadequate ultrafiltration, IQR = interquartile range, SD = standard deviation, UF = ultrafiltration.

We next explored whether intradialytic BPV reflected difficulty with volume management. For each patient, systolic and diastolic intradialytic BP variability (defined as the within-session standard deviation of repeated BP measurements) were averaged across both dialysate sodium phases and correlated with markers of volume stress. Higher systolic BP variability showed only a modest, statistically nonsignificant positive association with ultrafiltration shortfall (ρ = 0.36, *P* = .304) and a moderate, nonsignificant association with interdialytic weight gain (ρ = 0.52, *P* = .128). Associations between BP variability and intradialytic weight loss were weaker (ρ = 0.30, *P* = .405), and diastolic BP variability did not meaningfully track ultrafiltration shortfall (ρ = −0.23, *P* = .528). These data suggest that short-term hemodynamic lability during dialysis is not explained solely by volume status or fluid removal efficiency in this small, clinically stable cohort (Table 3).

**Table 3:** Association between intradialytic BPV and volume-related measures (Spearman correlations, N = 10).

Association tested	Spearman ρ	*P*-value
Intradialytic SBP variability versus ultrafiltration shortfall (%)	0.36	.304
Intradialytic diastolic BP variability versus ultrafiltration shortfall (%)	–0.23	.528
Intradialytic SBP variability versus interdialytic weight gain (% dry weight)	0.52	.128
Intradialytic SBP variability versus intradialytic weight loss (% dry weight removed)	0.30	.405

Intradialytic BP variability was defined as the within-session standard deviation of repeated systolic or diastolic BP measurements during each hemodialysis treatment. Ultrafiltration shortfall (%) = proportion of the prescribed ultrafiltration volume not achieved. Interdialytic weight gain (%) = [(pre-hemodialysis weight − previous post-hemodialysis weight)/ dry weight] × 100. Intradialytic weight loss (%) = [(pre-hemodialysis weight − post-hemodialysis weight)/ dry weight] × 100. For each patient, session-level values were averaged across both dialysate sodium phases (140 and 138 mmol/L) to derive a single patient-level summary. Correlations were assessed using Spearman rho; all analyses are exploratory, and *P*-values are unadjusted.

BPV = blood pressure variability, SBP = systolic blood pressure.

## 6. Discussion

In this prospective, fixed-order cross-over study, we investigated the effects of 2 dialysate sodium concentrations (140 and 138 mmol/L) on intradialytic BPV and volume management indicators in hemodialysis patients. Specifically, we hypothesized that BPV is related to volume load; therefore, we assessed whether low-sodium dialysate was accompanied by indicators such as ultrafiltration deficiency, interdialytic weight gain, or intradialytic weight loss. We did not observe a significant relationship between intradialytic systolic and diastolic BP variability and volume management load indicators. This finding suggests that reducing dialysate sodium by only 2 mmol/L does not, on its own, alter hemodynamic stability or volume load.

The literature has shown that low dialysate sodium concentration may have a certain effect on IDWG; for example, a systematic review and meta-analysis reported that dialysate sodium < 138 mmol/L could reduce IDWG by approximately − 0.40 kg (95% CI − 0.50 to − 0.30; *P* < .001).^[28]^ On the other hand, in a more recent study evaluating the effect of reducing dialysate sodium on intradialytic BPV, no significant difference was found in BPV indices when comparing 137 versus 140 mmol/L dialysate sodium (e.g., 48 hours SBP-SD: 16.99 ± 5.39 vs 16.98 ± 4.33 mm Hg, *P* = .982).^[29]^ Similarly, in our study, we found a weak correlation between systolic BPV and ultrafiltration deficit at ρ = 0.36 (*P* = .304); the correlation with diastolic BPV was even weaker (ρ=−0.23, *P* = .528). Overall, our findings align with prior work, as small sodium differences have not been found to have a strong effect on BPV or volume load indicators. However, what is interesting in our study is that the expected strong association between BPV and volume load indicators was not observed. This suggests that BPV in hemodialysis patients cannot be explained solely by volume load, and that other mechanisms may also play a role.

Volume load and ultrafiltration success are important sources of hemodynamic stress in hemodialysis^[2,30]^; high interdialytic weight gain and inadequate ultrafiltration have been associated with increased BPV and cardiovascular risk, along with increased volume load.^[31,32]^ However, our findings showed no strong correlation between BPV and these volume management parameters. In this case, it is thought that BPV is affected not only by fluid management but also by factors such as vascular tone changes, osmotic variables, dialysate-plasma sodium gradient, and interdialytic sodium load. For example, a review emphasized that the dialysate sodium-plasma sodium gradient may be associated with endothelial function and vascular stiffness.^[33]^ Furthermore, large cohort studies have found an association between low dialysate sodium (≤138 mmol/L) and high mortality; however, the authors noted that this finding is limited to observational data.^[34]^ In this context, our study, which applied a fixed 2 mmol/L reduction in dialysate sodium in stable hemodialysis patients, did not show significant changes in BPV or volume load indicators; therefore, these data do not support a 1-size-fits-all approach to lower dialysate sodium. This suggests that dialysate sodium adjustment requires individualized assessment, and a uniform strategy may be insufficient.

These findings offer several important implications for clinical practice. First, BPV is considered an indicator of high cardiovascular risk in hemodialysis patients; for example, the literature has shown that BPV is associated with mortality and cardiovascular events.^[35,36]^ Our study indicates that BPV cannot be significantly altered by modifying dialysate sodium alone. Therefore, to improve hemodynamic stability, it may be necessary to reevaluate not only dialysate sodium but also fluid management, optimization of antihypertensive therapy, vascular access status, and automatic systems in dialysis machines (e.g., sodium modeling). Second, volume load management – such as interdialytic weight gain and ultrafiltration success – remains important, but its lack of a direct strong correlation with BPV should prompt us to question the simplistic approach that “high BPV automatically means high volume load.” Finally, individualized dialysate sodium adjustment (e.g., based on parameters such as the patient’s plasma sodium level, interdialytic weight gain, residual diuresis) rather than a fixed low-sodium strategy points the way toward clinical research.

The main limitation of the study is that it was conducted at a single center and the sample size was small (N = 10). This structure limits the transferability of the findings to different patient profiles and different center practices. The matched (within-subject) cross-over design reduces intra-subject variability and improves efficiency; however, with N = 10, only medium to large differences in intradialytic SBPV are expected to be detected (approximately 5–6 mm Hg). Small effect sizes (<3 mm Hg) cannot be reliably ruled out. Statistically nonsignificant results are insufficient to demonstrate the equivalence of the 2 dialysate sodium levels; effects below the detectable threshold may have been missed.

The order of administration was fixed (140 mmol/L followed by 138 mmol/L) and randomization was not performed. A fixed order raises the possibility of period effects and carryover effects. The rapid approach of sodium balance to the new level and the maintenance of fixed dry weight targets may reduce the apparent carryover effect; complete exclusion is not possible.

The limited follow-up to 2 consecutive periods does not allow for the assessment of the relationship between intradialytic BPV and long-term cardiovascular outcomes. Blood pressure monitoring is limited to measurements taken at specific intervals during the dialysis session using automated devices; continuous recording or ambulatory blood pressure monitoring was not applied.

Measurement frequency and method may reduce the sensitivity of variability calculations. Mechanistic interpretations are limited in the absence of biochemical measurements such as plasma osmolality and renin–angiotensin system markers. Antihypertensive dose adjustments were made in a small number of patients due to clinical necessity; these changes may have had a limited effect on intradialytic haemodynamics. Subgroup analyses (gender, vascular access, coronary artery disease) should be considered descriptive and not extrapolated, as they remained at n = 3 to 4 per cell.

These limitations make it difficult to conclude “no effect” in this study, which tested the short-term effect of a small dialysate sodium change (2 mmol/L); rather, it highlights the issue of “failure to rule out a small effect.” Designs with larger samples, multiple centers, randomization, and longer follow-up periods, when used in conjunction with continuous/ambulatory blood pressure monitoring and relevant biochemical measurements, may more clearly demonstrate the clinical effect of the 140 to 138 mmol/L adjustment.

## Author contributions

**Conceptualization:** Ferah Taran.

**Data curation:** Ferah Taran.

**Methodology:** Hayriye Sayarlioglu.

**Software:** Ferah Taran.

**Supervision:** Hayriye Sayarlioglu.

**Writing – original draft:** Ferah Taran.

**Writing – review & editing:** Hayriye Sayarlioglu.
